# Morphometric and microstructural characteristics of hippocampal subfields in mesial temporal lobe epilepsy and their correlates with mnemonic discrimination

**DOI:** 10.3389/fneur.2023.1096873

**Published:** 2023-02-14

**Authors:** Alicia Comino Garcia-Munoz, Yasser Alemán-Gómez, Rafael Toledano, Claudia Poch, Irene García-Morales, Ángel Aledo-Serrano, Antonio Gil-Nagel, Pablo Campo

**Affiliations:** ^1^Centre de Résonance Magnétique Biologique et Médicale-Unité Mixte de Recherche 7339, Aix-Marseille Université, Marseille, France; ^2^Connectomics Lab, Lausanne University Hospital (CHUV), Lausanne, Switzerland; ^3^Epilepsy Unit, Neurology Department, Hospital Ruber Internacional, Madrid, Spain; ^4^Epilepsy Unit, Neurology Department, University Hospital Ramón y Cajal, Madrid, Spain; ^5^Facultad de Lenguas y Educación, Universidad de Nebrija, Madrid, Spain; ^6^Epilepsy Unit, Neurology Department, University Hospital of San Carlos, Madrid, Spain; ^7^Department of Basic Psychology, Autonoma University of Madrid, Madrid, Spain

**Keywords:** hippocampal subfields, microstructural image analysis, mnemonic discrimination, pattern separation, temporal lobe epilepsy (TLE)

## Abstract

**Introduction:**

Pattern separation (PS) is a fundamental aspect of memory creation that defines the ability to transform similar memory representations into distinct ones, so they do not overlap when storing and retrieving them. Experimental evidence in animal models and the study of other human pathologies have demonstrated the role of the hippocampus in PS, in particular of the dentate gyrus (DG) and CA3. Patients with mesial temporal lobe epilepsy with hippocampal sclerosis (MTLE-HE) commonly report mnemonic deficits that have been associated with failures in PS. However, the link between these impairments and the integrity of the hippocampal subfields in these patients has not yet been determined. The aim of this work is to explore the association between the ability to perform mnemonic functions and the integrity of hippocampal CA1, CA3, and DG in patients with unilateral MTLE-HE.

**Method:**

To reach this goal we evaluated the memory of patients with an improved object mnemonic similarity test. We then analyzed the hippocampal complex structural and microstructural integrity using diffusion weighted imaging.

**Results:**

Our results indicate that patients with unilateral MTLE-HE present alterations in both volume and microstructural properties at the level of the hippocampal subfields DG, CA1, CA3, and the subiculum, that sometimes depend on the lateralization of their epileptic focus. However, none of the specific changes was found to be directly related to the performance of the patients in a pattern separation task, which might indicate a contribution of various alterations to the mnemonic deficits or the key contribution of other structures to the function.

**Discussion:**

we established for the first time the alterations in both the volume and the microstructure at the level of the hippocampal subfields in a group of unilateral MTLE patients. We observed that these changes are greater in the DG and CA1 at the macrostructural level, and in CA3 and CA1 in the microstructural level. None of these changes had a direct relation to the performance of the patients in a pattern separation task, which suggests a contribution of various alterations to the loss of function.

## 1. Introduction

Medial temporal lobe epilepsy (MTLE) is the most common and well-defined form of drug-resistant epilepsy with focal seizures in adults (1). It usually debuts between the ages of 4 and 16 years and is characterized by seizures originating on the medial structures of the temporal lobe, the hippocampus, and/or the surrounding structures (2). A recent multicenter MRI study reported that most patients with MTLE showed marked atrophy or volume reduction in the hippocampus (3). This is a typical feature of hippocampal sclerosis (HS), the most common pathological substrate of MTLE. HS implies cell loss, which can affect any subfield of the hippocampus and is accompanied by gliosis (4). The International League Against Epilepsy (ILAE) has proposed a classification of HS based on histopathological findings: type I, in which CA1 and CA4 present the most severe neuronal loss and gliosis; type II, in which the damage is mostly restricted to CA1; and type III, in which CA4 is the structure predominantly affected (2).

In most cases, this atrophy appears on the hippocampus ipsilateral to the location of the epileptic focus characterized through EEG and more rarely appears bilaterally, especially in those patients with epileptogenicity in the right hemisphere (3, 5). The clinical manifestations of epilepsy are different in the two groups, and patients with right unilateral MTLE-HS tend to have earlier disease onset and more frequent and longer lasting seizures (6). This might show possible dissimilarities in the vulnerability of both hemispheres, or it can suggest that the pathophysiology changes depend on the side of lateralization (7).

While it is not unusual for people with epilepsy to present memory problems, those cases with epilepsy arising from the medial temporal lobe are at a higher risk of forgetfulness (8). Interestingly, the degree of memory impairment correlates with the degree of overall hippocampal atrophy in patients with MTLE-HS (6). Similarly, the type of MTLE-HS [according to the ILAE classification (9)] may also influence memory impairment (10–14). In patients with type II MTLE-HS, in which neural loss is predominantly observed in CA1, it was reported that declarative memory was not impaired (12, 15). In patients presenting types I and III, there is also neuronal loss in the dentate gyrus (DG) and, to a lesser extent, in CA3, contributing to a more severe loss of mnemonic function, which practically disappears if the loss of granule cells is higher than 60% (16). Nonetheless, other authors have reported that while patients with type II MTLE-HS pathology showed better group memory performance than those with type I MTLE-HS, almost half of them exhibited moderate to severe impairment in episodic memory tests (17–20).

Although memory can fail for several reasons, mnemonic interference is considered a potential cause of forgetting. Interference is viewed as a competition phenomenon so that when we encode experiences sharing features in memory, they become more prone to confusion (21). The risk of forgetting increases as the number of similar traces increases (22). To reduce interference, the circuit architecture within the hippocampal complex performs certain computations, making memories for overlapping events less similar, the so-called pattern separation (PS). Accumulating evidence supports a critical involvement of specific hippocampal subfields, especially the dentate gyrus, in pattern separation computations (23–26). Nonetheless, highly similar memories can compromise the efficiency of these computations due to the creation of overlapping representations within CA3, which, during retrieval, leads to reduced mnemonic discriminability (27). Several studies on neurological patients with different conditions have shown impairment in the ability to discriminate between highly similar episodes, which was related to a shift in hippocampal network dynamics (28). We and others have previously reported that patients with MTLE-HS showed a reduced ability to discriminate between similar objects when compared with normal controls (29–32), which was modulated by the number of similar traces stored in memory (33). Increasing the number of previously stored events affected performance in patients with MTLE-HS only in situations characterized by increased interference (i.e., discrimination between studied and similar items) but not in situations of low interference (i.e., discrimination between studied and novel items). We hypothesized that those results could be accounted for by a disrupted pattern separation function due to the concomitant neuronal loss and gliosis in different hippocampal subfields associated with HS (34). Despite the differential contribution of hippocampal subfields to PS, there is a dearth of information regarding how the alteration in specific subfields could account for memory difficulties in patients with MTL-HS. In the current study, our objective was to test the hypothesis that the integrity of certain hippocampal subfields, DG and CA3, would be more associated with mnemonic discrimination performance. With this aim, we sought to characterize not only the atrophy (volume loss) but also the microstructural organization of the hippocampus at the subfield level by using multimodal MRI data from patients with MTLE-HS to reveal novel features of the hippocampal anomalies (35). Microstructural features will be described using four diffusion MRI-derived metrics obtained from the spherical mean technique (SMT), and hippocampus subfield volumes will be automatically computed from T1-weighted images. SMT is a two-compartment model for diffusion imaging to estimate microscopic characteristics separately within an intra-neurite compartment (composed of fine processes like dendrites and axons) and an extra-neurite compartment (composed of cell bodies and extracellular space), irrespective of fiber crossings and orientation dispersion. The intra-neurite compartment is described by the intra-neurite volume fraction (VF_INTRA_); the extra-neurite compartment is depicted by the extra-neurite mean diffusivity (MD_EXTRA_).

To our knowledge, there are no studies describing how changes in the hippocampal subfield microstructure are related to patients with memory functioning MTLE. The novel use of diffusion analysis by tissue compartment could help deepen our knowledge of the pathophysiology of the disease to a neuronal level (35) and to better understand how the damage in these structures is contributing to the mnemonic problems in these patients. Moreover, analyzing the volumetric and microstructural changes in the subfields of patients with left and right MTLE-HS using MRI could help us to confirm the different susceptibility patterns of the two groups.

## 2. Methods

### 2.1. Sample of the study

A total of 20 patients (10 men and 10 women) with MTLE-HS and predominantly unilateral sclerosis, 8 in the right hemisphere and 12 in the left hemisphere, were recruited from the epilepsy units of the Ramón y Cajal, Ruber Internacional and Clínico San Carlos Hospitals in Madrid, Spain (Table 1).

**Table 1:** Demographics from the population of the study.

**Demographic variables**	***N***	**Percent (%)**	**Mean**	**SD**
**Age**	20		43.4	12.8
**Gender**				
Male	10	50		
Female	10	50		
**Affected hemisphere**				
Right	8	40		
Left	12	60		
**Age of onset**				
< 10 years	4	20		
10–20 years	6	30		
>20 years	10	50		
**Frequency of the crises (per week)**				
0–1	10	50		
4	10	50		
**Anti-epileptic drugs consumed**				
1–2	12	60		
3–5	8	40		

Presenting a previous diagnosis of temporal epilepsy associated with predominantly unilateral HS and age between 20 and 60 years old were the inclusion criteria to participate in the study. The diagnosis was made by their referring physician based on radiological criteria and EEG localization of the epileptic focus. Participants provided written informed consent: The study was conducted in accordance with the Declaration of Helsinki and was approved by the local Ethics Committee.

### 2.2. Neuropsychological tests

A neuropsychological battery was administered to assess the general cognitive functioning of patients. These tests included tasks of semantic and phonological fluency, object naming (36), visual discrimination (37), and general mnemonic performance [selective verbal recall, free recall, and multiple-choice recall (38)]. No patient was discarded after performing these tests.

To determine the specific mnemonic pattern separation function, a visual mnemonic discrimination task adapted from the Mnemonic Similarity Task (MST) was administered (33, 39). This is a task very sensitive to PS and therefore very useful to study encoding and discrimination in healthy adults, as well as the behavioral impact of all those alterations that affect hippocampal function [reviewed in Stark et al. (40)].

To test PS specifically, interference is introduced in two ways: by increasing the similarity between distractors and images already seen at different levels [reviewed in Reagh and Yassa (41)] and by varying the number of items of each category that appear in the initial presentation of images.

#### 2.2.1. Task description

The stimuli and procedure were adapted from those reported by Konkle et al. in their Experiment 1 (42). Accordingly, 689 color images of common objects from various categories were randomly presented. Each image is presented in isolation on a white background for 2,000 ms, with a variable inter-stimulus interval of 300–500 ms. The participant can choose to rest for a few seconds at any time. As a novelty with respect to other studies, the number of items presented in each category is varied between 1, 4, and 8 images. Thus, the second form of mnemonic interference is introduced. Within this first presentation of images, the participant is asked to press the spacebar if they see any image repeated.

After 30 min, the participant is subjected to a forced recognition test. In this test, two images appear on the screen on a white background, one from the previously presented list (“old”) and an additional one. This second image can be a specimen of a different category (“new”) or a very similar specimen of the same category (“distractor”). The images appear for 1,000 ms each with an interval of 300–550 ms between them.

Finally, after the presentation of the pair of images, a red cross appears on the screen for 2,500 ms and the participant is instructed to press the “1” key if it is the first image they have seen before, and “2” if it is the second one. If several images of the same category have been presented in the initial phase, in the recognition phase only the first image that has appeared is used to study mnemonic discrimination.

For the analysis of this task, percentages of success are obtained for each individual in the two conditions of the recognition phase: on the one hand, trials in which participants have to discriminate between an item previously shown and a new item (variable O_N, of old vs. new); and on the other hand, trials in which participants have to discriminate between an item previously shown and a similar distractor (variable O_S, of old vs. similar). In addition, for each condition, there are three levels (1, 4, and 8), depending on how many items of each category of the tested item were shown in the encoding phase, that is, each subject has six hit rates: O_N with 1, 4, and 8 levels of interference and O_S with 1, 4, and 8 levels of interference.

The variable O_N would be a measure of general recognition memory, while the variable O_S would serve to monitor the good performance of mnemonic discrimination between very similar items (PS).

Following Konkle et al. (42), we always tested mnemonic discrimination for the first object presented from each category. This allows to ensure that potential effects are due to interference from subsequently presented stimuli (42).

### 2.3. MRI acquisition

High-resolution structural MR images were acquired at a 3+Tesla magnetic resonance scanner (PRISMA, Siemens Medical Solutions) located at the Ruber International Hospital in Madrid.

A T1-weighted image (T1w), a T2-weighted image (T2w), and a sequence of DWIs were acquired for each subject. The T1w was acquired in a sagittal plane using an MPRAGE (Magnetization-Prepared Rapid Acquisition Gradient Echo) sequence with echo time (TE) = 2.15 ms, repetition time (TR) = 2,400 ms, inversion time (TI) = 1,000 ms, flip angle = 8°, field of view (FOV) = 176 × 240 × 256 mm^3^, and voxel size = 1 × 1 × 1 × 1 mm^3^. T2-weighted images were also acquired in 3D with TE= 564 ms, TR = 3,200 ms, flip angle=120°, bandwidth = 750 Hz, turbo factor = 314, FOV = 176 × 240 × 256 mm^3^, and voxel size = 1 × 1 × 1 × 1 mm^3^.

Diffusion-weighted images were acquired using an *echo planar imaging* (EPI) sequence with multiband excitation, TE = 67 ms, TR = 3,200 ms, flip angle = 90°, BW = 1,488 Hz, multiband factor = 2, FOV = 230 × 230 × 100 mm^3^, voxel size = 1.8 × 1.8 × 1.8 × 1.8 mm^3^, and b values = 0, 700, 1,000, and 2,500 s/mm^2^. Eleven non-diffusion-weighted (b = 0 s/mm^2^) and 179 diffusion-weighted images were acquired along gradient directions distributed over a unit radius sphere (18, 58, and 103 images for b = 700, 1,000, and 2,500 s/mm^2^, respectively).

### 2.4. Image analysis

Brain segmentation was performed on the T1-weighted image using the SPM8 image processing package (Statistical Parametric Mapping v8, https://www.fil.ion.ucl.ac.uk/spm/) and the VBM8 (voxel-based morphometry) processing package (43).

#### 2.4.1. Hippocampus subfield segmentation

For each patient, the anatomical T1-weighted image was employed to parcellate the hippocampus using the FreeSurfer package (v6.0.0, http://surfer.nmr.mgh.harvard.edu) (44). The brain mask image obtained by FreeSurfer during the skull stripping step was replaced by the manually edited brain mask to avoid watershed errors during the skull stripping process and, indirectly, to obtain a higher accuracy during the surface reconstruction and subcortical parcellation. Each hippocampus was further subdivided, using the atlas-based parcellation approach developed by Iglesias et al. (45) in twelve different subfields (parasubiculum, presubiculum, subiculum, CA1, CA2/3, CA4, granular layer of the DG, hippocampal amygdala transition area (HATA), fimbria, molecular layer of the subiculum and CA, hippocampal fissure, and hippocampal tail). This method uses a generative model that adapts very well to images with different contrasts or to the use of both T1- and T2-weighted images (46). Using a probability atlas of the hippocampal formation and Bayesian inference, a segmentation map is obtained where the probabilities are translated into voxels with different intensities (47).

**Figure 1:**
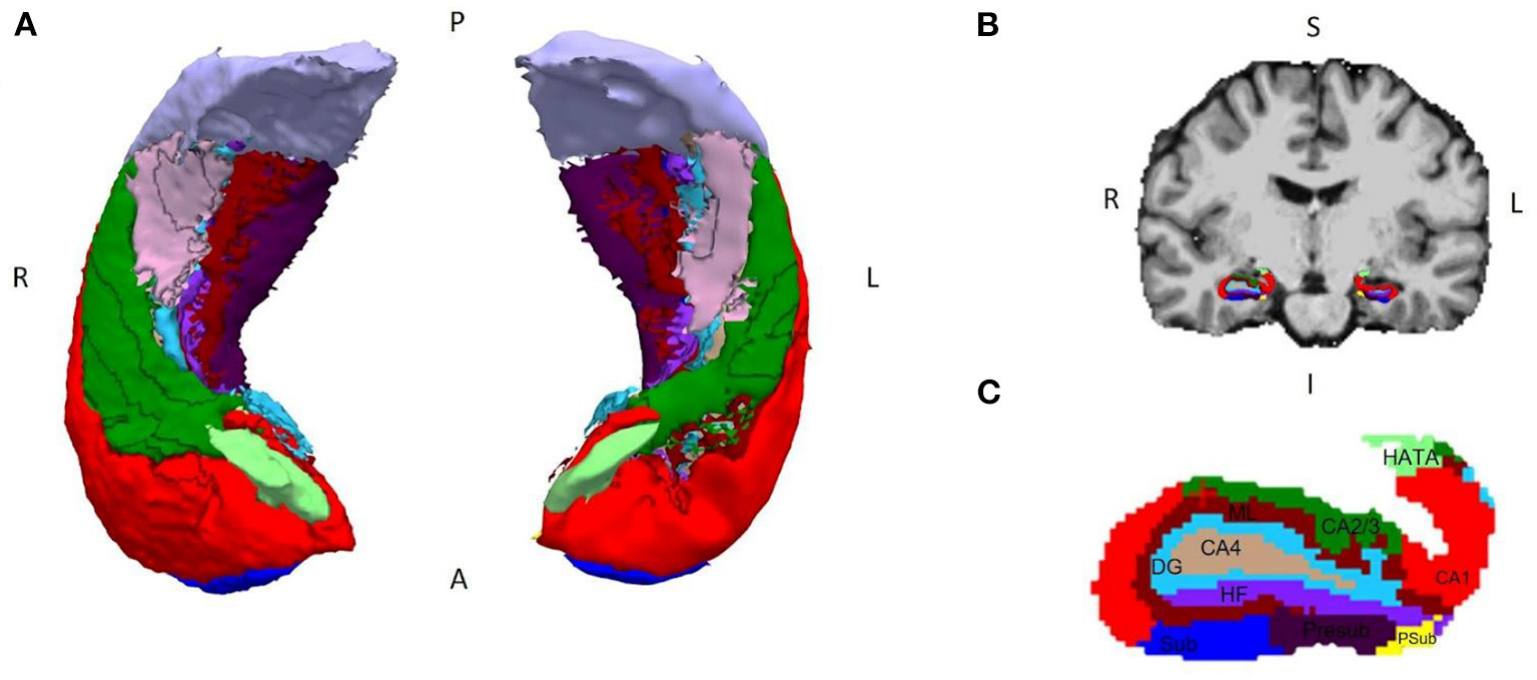
Segmentation of the hippocampal subregions made with FreeSurfer v.6.0.0. **(A)** 3D model of a bilateral hippocampus segmented in its axial view; **(B)** coronal view of the brain of a patient with MTLE-HS, with **(C)** the segmentation of the different subregions superimposed and augmented below (right hemisphere). Legend and color coding: subiculum (Sub, dark blue), CA1 (red), CA2/3 (green), DG (light blue), CA4 (light brown), parasubiculum (Psub, yellow), presubiculum (Presub, purple), HATA (light green), fimbria (pink), molecular layer of the subiculum and CA (ML, burgundy), hippocampal fissure (HF, violet), and hippocampal tail (gray). P, posterior; A, anterior; R, right; L, left; S, superior; I, inferior.

FreeSurfer labels as “DG” only the granule cell layer of this structure, since it appears with a much brighter contrast in MRI due to the granule cells being highly packed and it is easily differentiated. The polymorphic and molecular layers of the DG are not so easily differentiated because of their contrast and are included as part of other surrounding structures like CA4 or the molecular layer of the hippocampus. For this reason, in our analyses, the structure designated as DG refers only to the granular layer, whose cells have been shown to perform the neural computations necessary for PS (31). Figure 1 shows the result of a full hippocampal segmentation obtained for one of the patients included in the study.

To reduce the quantity of data, the hippocampal subregions finally used in the analysis were CA1, CA3, DG, and subiculum since these are the most affected in these patients according to the histopathological study from the ILAE (2). The volume of each of these regions was obtained from the parcellation of each of the subjects.

#### 2.4.2. Microstructure map computation

For each subject, an automatic quality control and image correction workflow was implemented and applied to its multi-shell DWI data. The workflow employed MRtrix3 (48) and FSL (49) processing tools for performing denoising, bias correction, intensity normalization, head motion correction (with gradient table rotation), eddy current, and distortion correction steps. A registration-based approach using Advanced Normalization Tools (ANTs) (50) was implemented to correct the geometrical distortion along the phase-encoding direction.

The corrected DWI dataset was used to estimate the diffusion tensor and different voxelwise microstructure maps by using the spherical mean technique methodology (51).

The diffusion tensor was employed to compute the fractional anisotropy (FA) and mean diffusivity (MD), while SMT was used to obtain the intra-neurite volume fraction (VF_INTRA_) and extra-neurite mean diffusivity (MD_EXTRA_) voxelwise scalar map.

For each subject, the mean value for each of these diffusion-derived metrics inside each hippocampal subfield was computed.

### 2.5. Statistical analysis

The data were analyzed using the Windows version of the SPSS 22.0 statistical package.

To study the mnemonic function, every patient's experimental score (O_S) was analyzed against their own control score (O_N) in the different interference conditions of the test (1, 4, and 8). The paired Wilcoxon signed-rank non-parametric test was used to analyze the change in the percentage of hits for each subject.

As all patients presented unilateral sclerosis, the contralateral hippocampus was used as a control region, comparing each subject with itself. Subsequently, the paired Wilcoxon signed-rank tests were used to analyze the volume variation and the diffusion variable (MD, FA, MD_EXTRA_, and VF_INTRA_) variation for each hippocampal subregion (CA1, CA3, DG, and the subiculum) of the hippocampus of the hemisphere ipsilateral to the epileptogenic focus (which we will refer to as “lesioned”) and contralateral to the focus (referred to as “non-lesioned”) in the patients.

The characterization of volume and diffusion variations between the lesioned and non-lesioned hemispheres was performed in two different ways: (1) for all patients as a whole, and (2) differentiating into two blocks according to the lesioned side (right or left hemisphere). To assess whether the volume and/or microstructure changes were different in both groups, we used the Kruskal–Wallis non-parametric test.

Finally, we explored the relationship between the scores in the mnemonic discrimination task in its two conditions and the volumes and diffusion variables of CA1, CA3, the DG, and the subiculum of the patients. For this, we used partial correlations and linear multiple regressions, controlling in each case for intracranial volume. The linear multiple regressions were done using the simultaneous analysis or *enter* method.

The results are expressed as mean ± SD or as a percentage. For all statistical tests, the significance level was set at alpha = 0.05 (*p* < 0.05 = ^*^; *p* < 0.01 = ^**^; *p* < 0.001 = ^***^).

## 3. Results

### 3.1. Mnemonic similarity task

**Figure 2:**
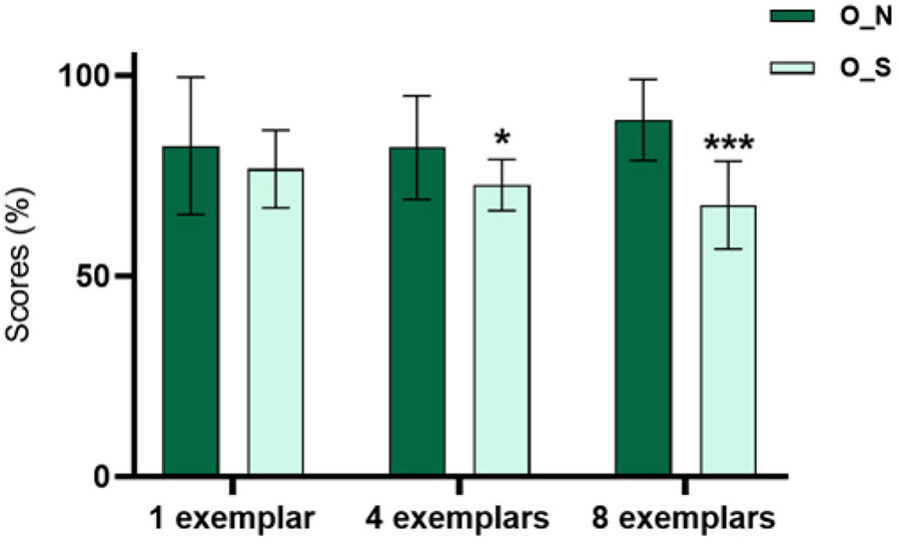
Relation between the scores in the different conditions of the MST for all patients (*n* = 20). The patients present a lower percentage of scores in the O_S condition with 4 (*p* < 0.05) and 8 (*p* < 0.001) examples of interference.

The patients presented a significantly lower mean score in conditions 4_O_S (72.68 ± 6.4) and 8_O_S (67.69 ± 10.94) when compared with conditions 4_O_N (82.01 ± 12.2) and 8_O_N (88.9 ± 10.12) (Figure 2).

Therefore, the patients had worse performance in the condition of the task that is specific for the PS than in the condition that quantifies the quality of the recognition memory. This is more accentuated in categories with higher levels of interference, as they require more resources to make the PS possible.

### 3.2. Characterization of volumetric alterations

#### 3.2.1. Whole sample

**Figure 3:**
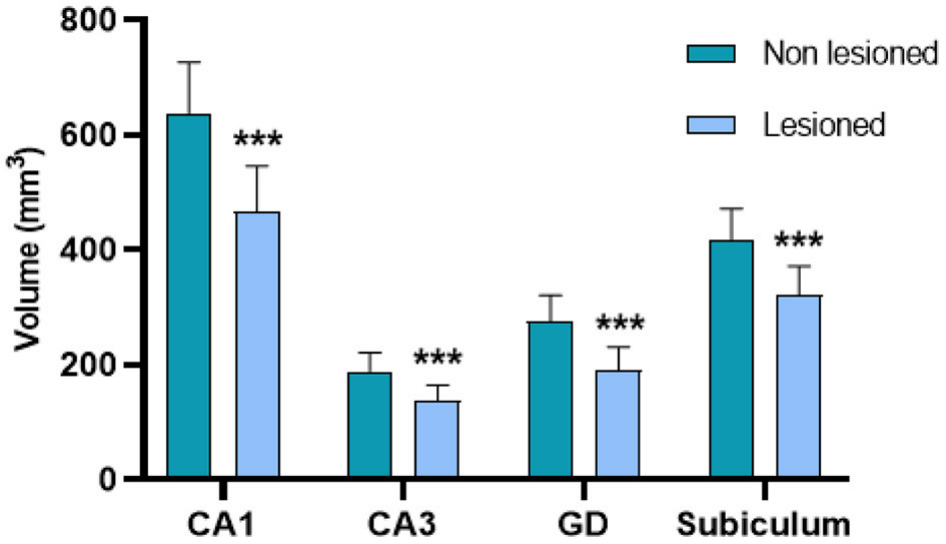
Volumetric changes in CA1, CA3, the DG, and the subiculum between the lesioned and non-lesioned hippocampi of the patients with MTLE-HS. There is a significant volume reduction in the four substructures (***p < 0.001).

**Figure 4:**
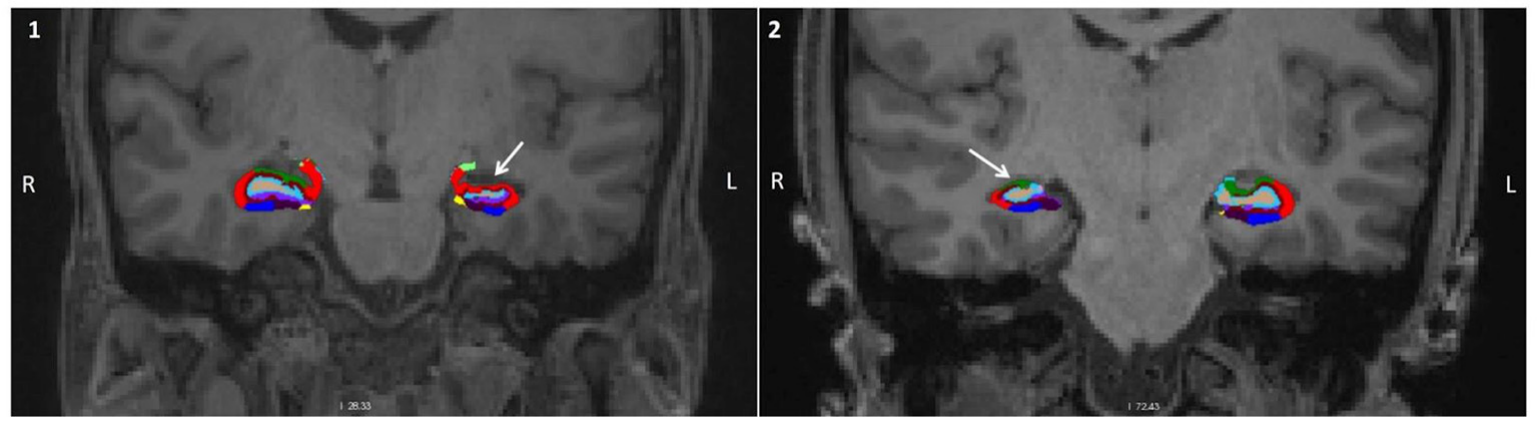
Characterization of the volumetric alterations. Coronal view of the brain of two patients with MTLE-HS with the hippocampal subregion segmentation superimposed. **(1)** corresponds to a patient with an epileptogenic focus on the left, and **(2)** to a patient with an epileptogenic focus on the right. In both cases, it can be observed a significant volume reduction in the hippocampus unilateral to the focus (white arrow) (red, CA1; green, CA3; light blue, DG; dark blue, subiculum).

The patients showed a mean reduction in total hippocampal volume of 864.62 ± 352.5 mm^3^. They also presented a reduced volume in all the analyzed subregions from the lesioned hemisphere (*p* < 0.001) compared to the contralateral analogous subfields. This reduction was bigger in DG (mean reduction of 31.7% of the volume compared with the non-lesioned hemisphere), being the most affected structure; and smaller in the subiculum (mean reduction of 22.83% of the volume), being the least affected structure (Figures 3, 4).

Using the volumetric data of their affected hippocampal substructures, we were able to classify them into the categories established by the ILAE (2). In 70% were considered Type I, having neuronal loss and gliosis predominantly in CA1 and CA4, and 30% were considered Type II, having neuronal loss mainly in CA1. None of the patients were considered Type III (Table 1). There was no relation between the patient's category and what side they had most affected.

#### 3.2.2. Lateralization analysis

We then analyzed whether volumetric changes were different between patients with the epileptic focus on the left hemisphere (L-MTLE) and patients with it on the right hemisphere (R-MTLE). The significant volume reduction between hemispheres, found in the whole sample analysis, was maintained for all substructures in the L-MTLE group (*p* < 0.01). In the R-MTLE, the reduction was significant for CA1 (*p* < 0.05), DG (*p* < 0.05), and subiculum (*p* < 0.05) but not for CA3 (*p* = 0.063). Moreover, the percentages of reduction in the subregions of the lesioned hemisphere were overall bigger in L-MTLE (Table 2).

**Table 2:** Mean values of the volumetric and diffusion parameters of the four analyzed substructures, separated by laterality type.

**Parameter**	**Laterality type**	**Structure**	**Mean (SD)**		**% change (Les vs. NonLes)**	**Pvalor (Les vs. NonLes)**
			**Lesioned**	**Non lesioned**		
Volume (m^3^)	RMTE	CA1	451.57 (100.26)	582.86 (95.86)	−22.6	**0.018**
		CA3	144 (38.77)	168.14 (28.56)	−14.36	0.063
		DG	188.28 (54.65)	249.14 (39.93)	−24.43	**0.018**
		Sub	290.57 (36.01)	380.57 (67.8)	−23.65	**0.018**
	LMTE	CA1	476.33 (69. 24)	667.08 (75.29)	−28.6	**0.002**
		CA3	132.58 (17.79)	199.25 (31.64)	−33.47	**0.002**
		DG	188.17 (36.02)	290.33 (44.2)	−35.2	**0.002**
		Sub	340.83 (49.36)	438.08 (37.9)	−22.2	**0.002**
MD	RMTE	CA1	0.00102 (0.000077)	0.00097 (0.000058)	5.41	0.093
		CA3	0.00116 (0.000117)	0.00100 (0.00009)	13.42	**0.017**
		DG	0.00103 (0.00005)	0.000911 (0.000034)	11.52	**0.012**
		Sub	0.00086 (0.000052)	0.00086 (0.000052)	4.16	0.069
	LMTE	CA1	0.00112 (0.00013)	0.00097 (0.000068)	13.19	**0.002**
		CA3	0.00123 (0.000099)	0.00109 (0.00014)	11.63	**0.002**
		DG	0.00109 (0.00014)	0.00093 (0.000061)	15.05	**0.028**
		Sub	0.00094 (0.00006)	0.00085 (0.000037)	9.48	**0.005**
FA	RMTE	CA1	0.16545 (0.02118)	0.13920 0.01751)	15.86	**0.05**
		CA3	0.15661 (0.06281)	0.15568 (0.04074)	0.6	0.889
		DG	0.14145 (0.04088)	0.113682 (0.02738)	3.28	0.674
		Sub	0.1986 (0.05175)	0.18107 (0.06322)	8.83	0.484
	LMTE	CA1	0.13535 (0.02004)	0.13567 (0.02263)	−0.24	0.06
		CA3	0.17711 (0.05296)	0.15746 (0.03420)	11.10	0.347
		DG	0.14606 (0.04367)	0.13620 (0.03059)	6.75	0.099
		Sub	0.15674 (0.02998)	0.14242 (0.02649)	9.14	0.638
MDEXTRA	RMTE	CA1	0.0015 (0.00013)	0.0014 (0.0001)	2.04	0.401
		CA3	0.0017 (0.00026)	0.0015 (0.00021)	8.97	**0.05**
		DG	0.0015 (0.00009)	0.0013 (0.00007)	10.8	**0.012**
		Sub	0.0013 (0.00006)	0.0012 (0.00004)	4.5	**0.036**
	LMTE	CA1	0.0015 (0.00018)	0.0015 (0.0002)	3.81	0.272
		CA3	0.0018 (0.00019)	0.0017 (0.00027)	3.85	0.272
		DG	0.0015 (0.0002)	0.0014 (0.00026)	2.21	0.388
		Sub	0.0013 (0.00014)	0.00013 (0.00011)	4.39	0.53
vINTRA	RMTE	CA1	0.32707 (0.02037)	0.35965 (0.00961)	−9.06	**0.017**
		CA3	0.30332 (0.61911)	0.35423 (0.04057)	−14.37	**0.05**
		DG	0.32052 (0.02541)	0.34553 (0.02301)	−7.24	**0.012**
		Sub	0.39117 (0.06460)	0.39435 (0.05212)	−0.8	1
	LMTE	CA1	0.32764 (0.04562)	0.33876 (0.06929)	−3.28	0.638
		CA3	0.35369 (0.0665)	0.36466 (0.05011)	−3.01	0.239
		DG	0.33248 (0.0556)	0.33646 (0.0519)	−1.18	0.388
		Sub	0.37007 (0.04665)	0.36459 (0.05964)	1.48	0.695

The percentage of change between the lesioned and non-lesioned hemispheres is indicated as positive if there is an increase in the value in the lesioned hemisphere and as negative when there is a reduction. RMTE, right medial temporal lobe sclerosis; LMTE, left medial temporal lobe sclerosis; MD, mean diffusivity; FA, fractional anisotropy; MDEXTRA, extra-neurite mean diffusivity; vINTRA, intra-neurite volume fraction. Bold values mean they are statistically significant.

To further standardize, we also established an asymmetry index for each patient, calculated as *(Non-Lesioned volume – Lesioned volume)/Non-Lesioned Volume*. The asymmetry indexes of CA1, DG, and subiculum were not different among the patients of the R-MTLE and the L-MTLE groups but presented a significantly different distribution for CA3 (*p* < 0.05).

#### 3.2.3. Relation with cognitive measures

To establish the possible relation between the volumetric changes and the performance in the MST, we correlated the volume asymmetry index (calculated as shown before) for each substructure and the scores on the different conditions of the test for each patient.

We found significant relations only among some of the task conditions that tested recognition memory (O_N). Specifically, there was a negative correlation between the asymmetry index of CA1 (R = −0.627, *p* < 0.005), CA3 (r = −0.666, *p* < 0.005), and the subiculum (R = −0.523, *p* < 0.01) and the results in the condition with a greater number of exemplars (8_O_N). There was also a trend in the same direction of the DG volume asymmetry, although it did not reach significance (R = −0.439, *p* = 0.06), as well as a positive correlation between the volume of CA3 and that same condition (R = 0.595, *p* < 0.05).

**Figure 5:**
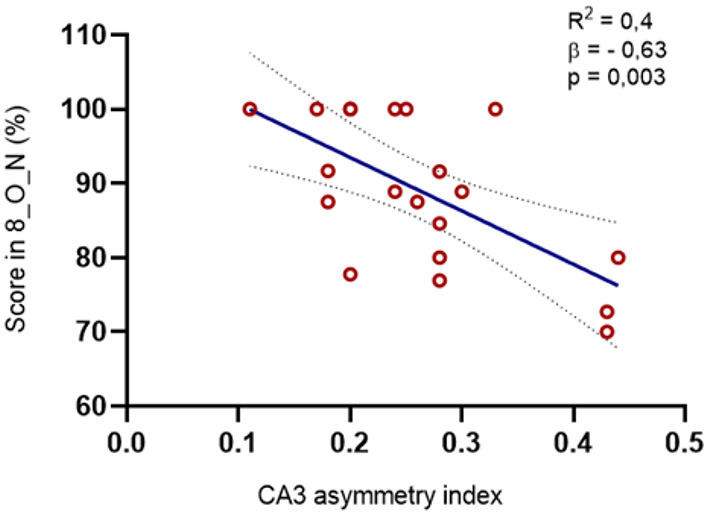
Relation between the asymmetry index in the volume of CA3 and the MST scores. We found a negative correlation between the CA3 asymmetry index and the percentage of right answers in the O_N condition at maximal interference levels (r = −0,666, *p* = 0,002): The regression model was also significative for this structure [R2 = 0.395, *F*_(1, 8)_ = 11.767, *p* = 0.003, β = −0.629].

These results were further confirmed with a linear regression model that showed that the asymmetry in the volume of CA3 between the lesioned and non-lesioned hemispheres is contributing to approximately 60% of the variation of performance in the 8_O_N condition (*p* < 0.01) (Figure 5).

### 3.3. Characterization of microstructure changes

#### 3.3.1. Whole sample

Similar to the results found for the volume, the microstructural features were affected in CA1, CA3, the DG, and the subiculum of the lesioned hippocampus compared to the values in the non-lesioned side.

**Figure 6:**
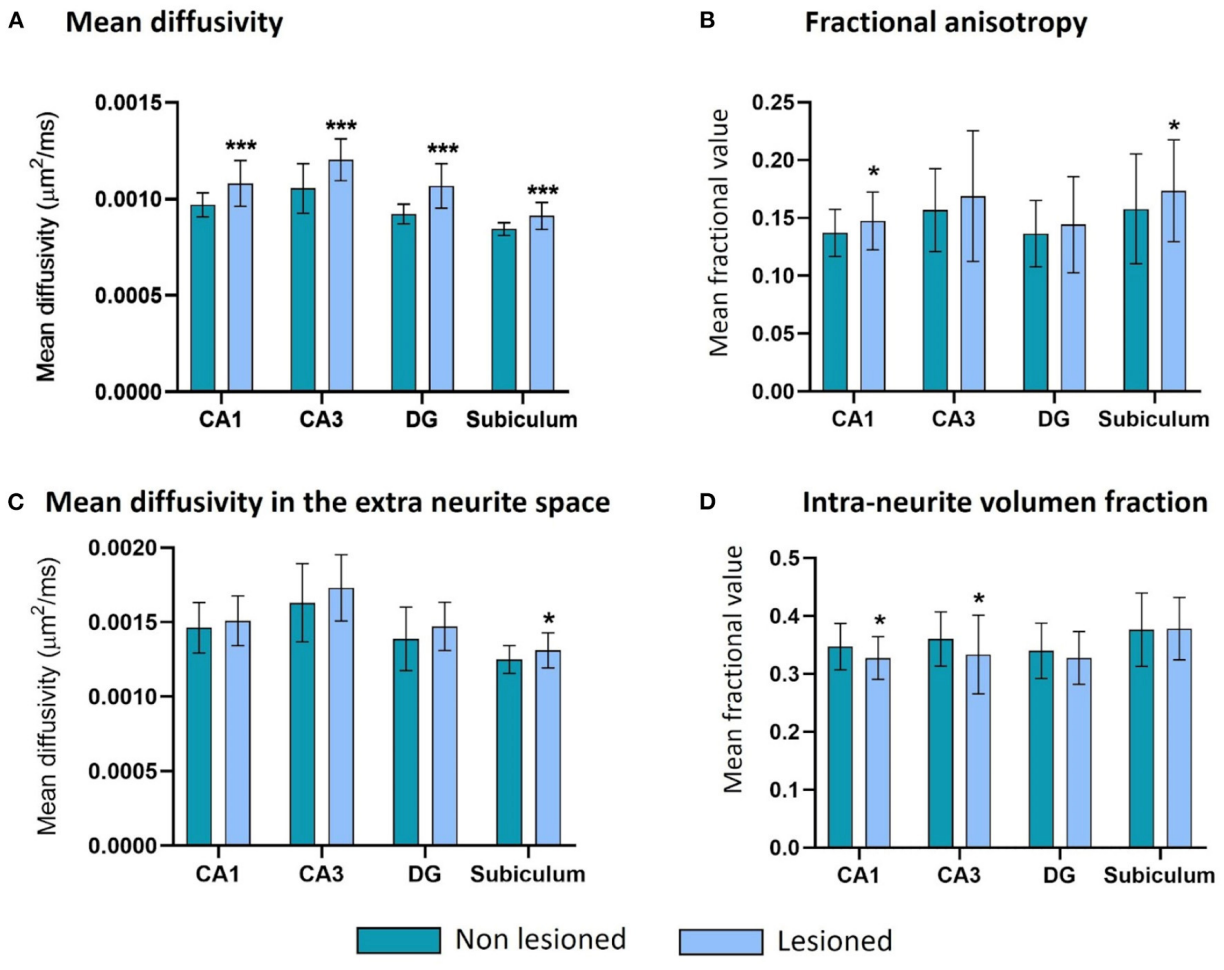
Microstructure changes in CA1, CA3, the DG and the subiculum contralateral and ipsilateral to the epileptogenic focus in MTLE-HS patients. There are significant differences (*** = *p* < 0.001) in all four structures for the MD **(A)**, in CA1 and the subiculum for the FA (* = *p* < 0.05) **(B)**, in the subiculum for the MDEXTRA (* = *p* < 0.05) **(C)**, and in CA1 and CA3 for the VFINTRA (* = *p* < 0.05) **(D)**.

The rise in MD was significant for all the structures (Figure 6A) but was proportionally bigger in the DG (an average of 16% more MD was observed in the lesioned structures, in comparison with an 11, 14, and 8% rise in CA1, CA3, and the subiculum, respectively). The MD in the extra-neurite space (MD_EXTRA_) was also bigger in the lesioned structures, but the changes only reached significance in the subiculum (Figure 6C). These changes could suggest damage in the gray matter (GM) at the level of the neuronal somas and the glia.

There were also significant changes in the parameters related to the white matter (WM) of the lesioned hippocampus. The mean fractional anisotropy (FA) was bigger in the lesioned CA1 and subiculum (Figure 6B), and the intra-volume fraction (VF_INTRA_) was significantly lower in CA1 and CA3 (Figure 6D).

The DG shows the same tendencies in both parameters but the differences between hemispheres do not reach significance.

#### 3.3.2. Lateralization analysis

The L-MTLE group did not present significant differences in the parameters MD_EXTRA_, FA, or VF_INTRA_ in any of the four analyzed subregions, but the rise of MD in the lesioned hippocampus was still maintained just as it happened in the whole group analysis.

The R-MTLE group, however, did only present these significant increases in MD in CA3 and the DG. For the other three parameters, we found changes in this group that were not present in the L-MTLE one. These included a raised MD_EXTRA_ in the subiculum, CA3, and DG, as well as FA in CA1, and decreased VF_INTRA_ in CA1, CA3, and the DG (Table 2).

Asymmetry indexes were also calculated for these parameters in the same way as for the volume ones. There were significant differences (*p* < 0.05) between the asymmetries in the two groups for two parameters, both related to the WM: VF_INTRA_ of CA3 and FA of CA1.

#### 3.3.3. Relation with cognitive measures

Similar to the volume results, we found no significant correlations between the microstructural changes in the hippocampal subfields of our group of patients and their scores in the mnemonic similarity task.

**Figure 7:**
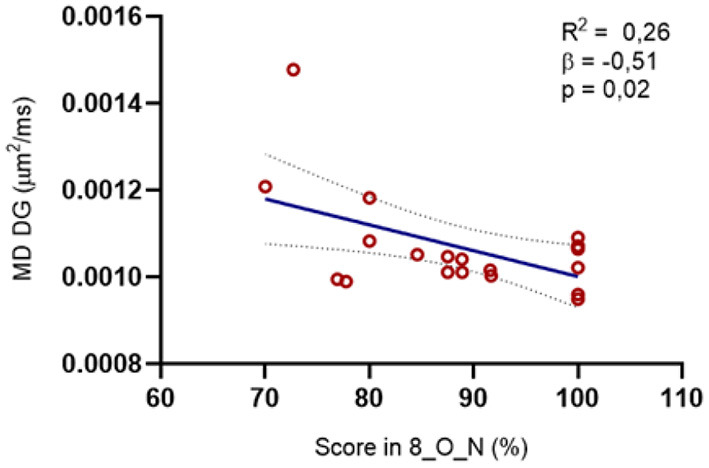
Relation between the microstructure of the DG in the lesioned hemisphere and the MST scores. We found a negative correlation between the MD on the DG and the percentage of right answers in the O_N condition at maximal interference levels (R = −0.512, *p* = 0.02): The regression model was also significative for this structure [R2 = 0.264, *F*_(1, 8)_ = 6.455, *p* = 0.02, β = −0.514].

However, we did find a negative correlation between the MD of the lesioned DG and the scores in the mnemonic discrimination 8_O_N condition (R: −0.512, *p* = 0.025). Likewise, we observed a tendency when we explored the MD of CA1 (R: −0.452, *p* = 0.052) and the subiculum (R: −0.455, *p* = 0.05). When we plot these results in a regression model, we can see that the MD of the lesioned GD is contributing to approximately 50% of the variance in the performance on the 8_O_N condition (*p* < 0.05) (Figure 7).

## 4. Discussion

In the current study, we obtained the morphometric and microstructural MRI data of each of the hippocampal subfields in a group of patients with MTLE-HS. In addition, this group of patients performed a modified version of the classic MST task (52) that had been previously developed by our group (33, 39). We have shown that patients with MTLE-HS had a lower percentage of correct answers than controls in the PS-specific part of the test (i.e., discrimination between studied and similar items), especially in the categories with the highest level of interference (4 and 8 items presented). On the contrary, in the part of the MST task assessing general recognition memory (i.e., discrimination between studied and novel items), their performance did not differ from controls (33). The aim of our study was to characterize microstructural abnormalities in each of the hippocampal subfields in these patients (35) and, for the first time, to determine the relation of these metrics with their mnemonic performance on a behavioral pattern separation task.

Regarding the volumetric alterations in the hippocampal substructures, the patients present less volume in the four hippocampal substructures (CA1, CA3, DG, and subiculum) ipsilateral to the epileptogenic focus. Of these, the DG and CA1 are the structures with the greatest reduction with respect to their counterparts in the contralesional hippocampus. Other structural MRI studies have also identified these two substructures as the most affected in this disease (53, 54), also when compared with patients with non-epileptic controls and non-HS MTLE (53, 55, 56). This is also consistent with data from the ILAE histopathological classification study of MTLE-HS. According to this study, the most common type of HS is with severe neuronal loss in the CA1 and GD-CA4 regions (2), which would be confirmed in our group of patients, where 70% of them were classified as type I (Table 1).

At the microstructural level, the most representative change is found at the mean diffusivity level. MD is a parameter that allows us to determine tissue integrity, since with cell death, axonal degeneration, and gliosis, the barriers that retain water are broken down and water diffuses more freely through the tissue (57). Consistent with the macroscopic data, MD increases in all the subregions, suggesting damage at the cellular level. This damage would be greatest in the DG, which is postulated to be the most affected structure in MTLE-HS (58). A recent study reported similar results (35).

In addition, this study introduces as a novelty the separate analysis of two tissue compartments: the set of neurites (dendrites and axons) on the one hand and the neuronal bodies, the glia, and the extracellular space on the other. This differentiation allows us to specify at which cellular level the damage appears, which could otherwise just be observed as macroscopic atrophy. When analyzing the changes in water diffusion in the two compartments, we found a tendency toward an increase in water diffusion in the extra-neurite space, accompanied by a decrease in the volume fraction of water present in the intra-neurite compartment. These changes occur especially in CA1 and CA3. In other words, it is possible that damage is occurring at the level of the WM fibers that would cause water to escape into the GM, where it also diffuses more freely. This pattern of change could indicate an alteration in axonal density or be secondary to a reduction in the myelin sheath, which would favor the outflow of water from the fibers (59). For poorly myelinated or unmyelinated axons, it is more difficult to differentiate between hypomyelination and reduced axonal density, and further histopathological tests would be necessary to corroborate specific alterations in the myelin sheath of axons (60). We also observed that the MD in the extra-neurite space (MDEXTRA) was also bigger in all the subfields of the lesioned hippocampus, but the changes only reached significance in the subiculum. This specific alteration in the subiculum could be related to the increased excitability of the subiculum observed in patients with TLE and in animal models of TLE (61–64). Interestingly, Prada Jardim et al. (13) observed that postsurgical decline in memory at 1 year was associated with degeneration in the subiculum.

Based on a recent study by Winston et al. (57), it seems more probable that these observed changes are associated with reduced neurite density (65) than with the fraction of water in the myelin sheath, as they found a positive association between the first parameter and the increased MD in the GM of the ipsilateral temporal lobe.

Notably, changes observed in the group as a whole were not always maintained when we separated the data by the hemisphere in which the epileptic focus was present. When taking into account lateralization, there has historically been no agreement on which group is more severely affected, with studies stating that the cortical thickness and WM connectivity are more compromised in those with a focus on the left hemisphere (66, 67) and others claiming that those with the epileptogenic zone on the right hemisphere are more affected (7, 55). There is also no consensus on a possible association between the pattern of atrophy and memory performance in these two groups (30). Our results are in agreement with a recent study by Moghaddam et al. (7) that stated that L-MTLE presented more asymmetry between the two hemispheres than R-MTLE when comparing only the two groups. We add that the difference is seen especially in CA3, which is not reduced when comparing the two hemispheres in R-MTLE. However, with these data, we cannot affirm that the patients with R-MTLE have less HS. On the contrary, it is highly probable that these results are due to the right-sided patients being more prone to bilateral sclerosis. In this sense, it seems that sclerosis would affect the contralateral structures more significantly, especially CA3, which would explain these particular results in the intra-patient comparison.

However, if we look at the microstructure results, the pattern that emerges shows that the L-MTLE group has almost no significant differences in any of the parameters on the four subregions, while the R-MTLE group does present some significant microstructure changes. These changes have to do mostly with the FA and the intra-neurite fractional volume, that is, with the WM parameters.

Therefore, while when we look at macroscopic data the left-sided group is more asymmetric, from a microstructure point of view, it is the right-sided group that presents more differences between hemispheres. In summary, we cannot confirm which group of patients is more affected by the disease; however, our results suggest that they are differentially affected, especially at a microstructural level. This difference probably has to do with changes in the WM that might be not equally damaged due to the differential patterns of onset and progression of the disease (6).

Finally, we studied the possible relation between the alterations observed in certain hippocampal regions with MRI and the scores in the MST obtained by each patient. In our study, it is the volumetric difference between CA3 in the lesioned and non-lesioned hemispheres which was able to better predict the performance in recognition memory (68). In terms of diffusion parameters, this performance is also better predicted by the level of alteration of the MD in the ipsilateral DG. However, despite our initial hypothesis that a disrupted pattern separation function could be due to the concomitant neuronal loss and gliosis in different hippocampal subfields associated with HS (10, 34), none of the observed alterations in volume or diffusion was associated with the performance in the pattern separation part of the cognitive task (69). This happened for all the studied substructures, both in the lesioned and non-lesioned hemispheres. These results are in agreement with those from the study of Usugi et al. (70), who did not find any significant associations between pattern separation ability and subfield volumes in healthy subjects. They concluded that the idea that “bigger is better,” that the larger the volume of a structure the better it is able to perform its function, does not apply in the case of pattern separation and the hippocampal subfields. This lack of a direct relationship between the size of the hippocampal subfields and PS function is also seen in our study with patients with MTLE. Recently, Grupe et al. (71) did not find a hypothesized correlation between DG/CA3 volume and behavioral pattern separation measures. Furthermore, our results are also extended to the microstructure parameters, concluding that the damage of one specific hippocampal part or the alteration of certain diffusion parameters cannot, on their own, explain the impairment that these patients present when performing pattern separation. Although Dillon et al. (68) found that DG fractional volume predicted accuracy and speed of pattern separation, they found that neither other hippocampal subfields nor hippocampal WM predicted pattern separation performance. In addition, there is evidence that mnemonic discrimination relies on other brain regions, particularly the dorsal medial prefrontal cortex (72). In this sense, two recent studies suggest that multifactorial variables are likely to underlie the memory impairment associated with MTLE-HS. Prada Jardim et al. (13) were unable to observe any association between memory performance and neuropathological subtypes of hippocampal sclerosis either pre- or post-operatively [see also (14)]. Similarly, Lalani et al. (29) also indicated that there were no differences in the magnitude of pattern separation impairment in TLE with and without HS.

The main limitation of our study is the modest sample size. To mitigate the effect of individual variability and to consolidate the results obtained, it would be advisable to replicate the analyses in a larger cohort.

Another limitation is the current interpretation of the results of the diffusion parameters. Although several studies have linked changes similar to those described by us to specific conditions (such as increased extra-neurite diffusion together with a reduction in intra-neurite water volume, which have been linked to myelin sheath reduction (59), it is not possible to ascertain the specific nature of these changes with structural MRI data alone. FA, for example, is a very sensitive marker of WM neuropathology but also very non-specific (73, 74). The augmentation of the FA in CA1 or the subiculum shown in this study indicates a better alignment of WM fibers, which kind contradicts the logical explanation of damage in the neuronal tracks. However, it may be the result of an increase in axonal density, but it may also indicate that the fiber crossings have degenerated and the remaining fibers give more anisotropic data because they have similar orientations (74). In addition, the hippocampus is a substructure composed mainly of GM, making it more difficult to determine specific WM alterations. By using analysis techniques such as SMT, we can more accurately determine parameters related to the WM, which is present to a lesser extent in the hippocampus. However, the technique still needs to be further refined and combined with other post-mortem or postsurgical tissue analysis techniques to confirm the specific nature of the alterations.

The very nature of MTLE-HS introduces a limitation in our results, as it has a very localized unilateral onset, but sclerosis tends to spread to other brain regions both ipsilateral and contralateral (74). Furthermore, physiologically, the right hippocampus tends to have a larger volume than the left (75). This asymmetry is mostly observed in the DG and CA3 and to a lesser extent in CA1 (76). In this study, we have chosen patients with a predominantly unilateral epileptogenic focus to compare the more affected hemisphere with the less affected hemisphere. However, in the absence of data on their pre-sclerosis status, we cannot be sure that the uninjured hippocampus is completely healthy or that the observed structural differences are not due to pre-disease inequalities. This may be a bias when analyzing, especially the asymmetry indices.

In this study, we established for the first time the alterations in both the volume and the microstructure at the level of the hippocampal subfields DG, CA1, CA3, and the subiculum in a group of patients with unilateral MTLE. We observed that these changes are greater in the DG and CA1 at the macrostructural level and in CA3 and CA1 at the microstructural level. None of these changes had a direct relation to the performance of the patients in a pattern separation task, which suggests a contribution of various alterations to the loss of function. Finally, we further confirmed that these patients are differentially affected depending on the lateralization of their epileptic focus. These differences are probably related to the degree of affection of the WM, which would help explain the dissidence in clinical manifestations and disease development between the two groups.

## Data availability statement

The raw data supporting the conclusions of this article will be made available by the authors, without undue reservation.

## Ethics statement

The studies involving human participants were reviewed and approved by Dirección del HURyC. The patients/participants provided their written informed consent to participate in this study.

## Author contributions

PC: conceptualization, funding acquisition, investigation, project administration, supervision, validation, and writing. AC: data curation, formal analysis, investigation, and writing. YA-G: formal analysis, investigation, methodology, software, supervision, and writing. RT: data curation, funding acquisition, investigation, and writing. CP: investigation, methodology, and writing. ÁA-S and IG-M: data curation, investigation, and writing. AG-N: data curation, investigation, supervision, and writing. All authors contributed to the article and approved the submitted version.
